# Nursing Workforce Preparedness and Resilience in Health Emergencies (2000–2025): A Bibliometric Review of Thematic Evolution and Emerging Hotspots

**DOI:** 10.1155/jonm/7758497

**Published:** 2026-07-29

**Authors:** Walton Wider, Muhammad Aledeh, Laís Cibely Dos Reis Prazeres Feitosa, Erica Jeronimo Da Costa, Thiago F. Santos, Alex S. Borromeo

**Affiliations:** ^1^ Faculty of Business and Communications, INTI International University, Nilai, Negeri Sembilan, Malaysia, newinti.edu.my; ^2^ Department of Psychiatry, Wiener Gesundheitsverbund, Klinik Donaustadt, Vienna, Austria; ^3^ Universidade do Grande Rio (UNIGRANRIO Afya), Duque de Caxias, Rio de Janeiro, Brazil; ^4^ Centro Universitário FACEX (UNIFACEX), Natal, Rio Grande do Norte, Brazil; ^5^ Postgraduate Program in Chemical Engineering, Technology Center, Federal University of Rio Grande do Norte, Natal, Rio Grande do Norte, Brazil, ufrn.br; ^6^ Department of Nursing, College of Nursing, Bulacan State University, City of Malolos, Bulacan, Philippines

**Keywords:** bibliometric analysis, disaster preparedness, health emergencies, nursing workforce, workforce preparedness

## Abstract

**Aim:**

To map research on nursing workforce preparedness and resilience in health emergencies from 2000 to 2025 and propose an integrated framework.

**Design:**

Bibliometric review with science mapping and thematic evolution.

**Methods:**

Scopus was searched for journal articles and reviews on nursing workforce preparedness and resilience in health emergencies published from 2000 to 2025. Records were screened using year, language, document type, duplicate title, and metadata‐based scope criteria. Co‐citation and co‐word analyses were conducted to map intellectual foundations and conceptual structures. Time‐sliced analysis examined thematic evolution across 2000–2009, 2010–2019, and 2020–2025, while emerging hotspots were identified using keyword growth and recency indicators.

**Results:**

The final retained corpus included 7570 documents, composed of 6950 articles and 620 reviews. Publication output showed a marked post‐2020 surge. Co‐citation analysis highlighted intellectual foundations related to disaster nursing competence, psychological resilience and mental health, leadership, staffing, safety, and workforce systems. Co‐word analysis indicated major domains of clinical competence, emergency and acute‐care delivery, pandemic‐related psychological burden and resilience, workforce organization and emergency service capacity, and nursing education for disaster preparedness. Emerging hotspots included burnout, moral distress, resilience, telemedicine, mental health, and qualitative inquiry.

**Conclusion:**

Nursing workforce preparedness research has evolved into a multidimensional, system‐oriented agenda that integrates competence, resilience, and structural readiness.

**Implications for Nursing Management:**

Nurse managers should treat emergency preparedness as a strategic workforce‐management responsibility, not only as a clinical or disaster‐planning task. The findings support competency‐based preparedness training, workforce policies for surge staffing and role deployment, organizational capacity building, mental health and resilience supports, leadership preparation, safety infrastructure, and data‐informed preparedness planning to sustain nursing performance during future health emergencies.

## 1. Introduction

Health emergencies repeatedly stress‐test the nursing workforce through surges in patient volume and clinical complexity and, critically, through sustained psychological strain, moral distress, and occupational risk. During COVID‐19, rapid increases in cases and system pressures, following the World Health Organization’s declaration of COVID‐19 as a global pandemic in early 2020 [1], renewed global attention to protecting and sustaining healthcare workers [2] and to the psychosocial consequences of outbreak‐control measures such as quarantine [3]. Empirical studies from the pandemic period documented substantial mental health burdens among frontline healthcare workers, including anxiety, depression, insomnia, and trauma‐related outcomes [4, 5], alongside calls to strengthen mental healthcare supports for medical staff [6]. Among nurses, reviews highlighted frontline experiences, burnout, and occupational stressors during COVID‐19 [7, 8], reinforcing that preparedness cannot be reduced to logistics alone but must include human capacity, well‐being, and sustained adaptive functioning under prolonged disruption.

Conceptually, nursing workforce preparedness is grounded in competence frameworks that define what nurses should know and be able to do across the disaster cycle. Early work emphasized core competencies for emergency and disaster preparedness across nursing roles [9]. These were later complemented by global and educational frameworks: the International Council of Nurses articulated disaster nursing competencies [10] and expanded these into updated core competencies [11], while nursing education standards have evolved to define professional competency expectations [12, 13]. However, competence alone does not ensure sustained performance during crises. The construct of resilience—commonly defined as the capacity to maintain or regain functioning under adversity—has been widely operationalized in health research through validated measures such as the Connor–Davidson Resilience Scale [14, 15] and clarified in nursing‐focused concept analyses [16]. In health emergencies, competence and resilience intersect: competence supports safe and effective practice, while resilience supports endurance, recovery, and workforce retention under extreme conditions.

At the systems level, preparedness and resilience are inseparable from workforce structures such as staffing adequacy, work environments, leadership, and organizational support. Foundational evidence links nurse staffing to patient outcomes and to nurse burnout and job dissatisfaction [17], with multicountry studies further demonstrating the role of staffing and education in determining mortality outcomes [18]. During emergencies, workforce resilience is shaped by willingness to work, deployment policies, and perceived preparedness—issues repeatedly examined in disaster contexts [19–22]. Leadership has likewise been emphasized as critical, positioning nurses as central leaders in preparedness and response [23], and global reports highlight investment in education, jobs, and leadership as essential for strengthening nursing worldwide [24]. Collectively, the literature is extensive but fragmented across hazards, levels of analysis (individual, organizational, and policy), and outcomes (competence, mental health, staffing, and education).

Despite this expanding evidence base, most syntheses remain dominated by topic‐specific reviews rather than a unified, longitudinal perspective on how the knowledge base has evolved. Reviews have advanced understanding of disaster preparedness among nurses [25] and competencies and psychological preparedness for disasters [26]. Integrative reviews synthesized resilience among healthcare workers during COVID‐19 [27] and clarified nurse resilience as a concept [16]. While essential, these approaches often focus on narrower windows or single hazards, privileging narrative synthesis over field‐level structure and offering limited insight into how emphases shift across major global health events and policy eras.

A bibliometric approach is suited to address these limitations by mapping productivity, influence, collaboration patterns, and co‐occurring concepts at scale, thereby revealing the intellectual structure of a field and how it changes over time [28, 29]. This is particularly valuable for nursing workforce preparedness, where “health emergencies” encompass pandemics, outbreaks, and disasters that generate distinct research traditions, ranging from competency‐based preparedness studies [30–32] to pandemic‐driven workforce mental health and mitigation scholarship [3–6].

Accordingly, this study aims to provide a comprehensive, longitudinal synthesis of scholarship on nursing workforce preparedness, readiness, capacity, competence, and resilience across health emergencies (2000–2025). Specifically, we (1) characterize publication growth, outlets, and collaboration patterns; (2) identify influential sources and intellectual foundations; (3) map dominant conceptual clusters; (4) examine thematic evolution across three phases (2000–2009; 2010–2019; and 2020–2025); and (5) detect emerging hotspots. The novelty lies in integrating time‐sliced thematic evolution, quantified hotspot detection, and a three‐pillar preparedness model synthesizing competence/readiness, resilience/mental health protection, and system‐level supports [11, 23, 24].

## 2. Methods

### 2.1. Bibliometric Analysis

This study employed bibliometric analysis to examine and map the intellectual and conceptual structure of scholarship on nursing workforce preparedness and resilience in health emergencies. Bibliometric methods analyze patterns in publications and citations to identify influential works, contributors, and dominant themes and to visualize how a field develops over time [28, 29].

To capture both foundational knowledge and thematic evolution, two complementary bibliometric techniques were applied:

i. Co‐citation analysis: Co‐citation analysis identifies intellectual foundations by examining how frequently pairs of documents are cited together in later publications. The core assumption is that repeatedly co‐cited documents share conceptual, theoretical, or methodological relationships [33]. This approach is widely used to uncover seminal publications and enduring frameworks that shape a research domain [29, 34].
ii. Co‐word analysis: Co‐word analysis maps conceptual structure by examining patterns of keyword co‐occurrence across titles, abstracts, and keyword fields. It assumes that terms frequently appearing together represent closely related concepts [35]. Co‐word analysis is useful for identifying dominant themes, emerging topics, and shifts in research focus over time [29, 36].

### 2.2. Search Strategy and Data Collection

A structured bibliometric search was conducted in Scopus on 27 December 2025. The search used the TITLE‐ABS‐KEY field and combined three mandatory concept blocks: (1) nursing‐related terms, (2) preparedness‐related constructs, and (3) health emergency contexts (see Table 1). The final search string was as follows:

 TITLE‐ABS‐KEY (nurse^∗^ OR “nursing workforce” OR “nursing profession”) AND
 (preparedness OR readiness OR resilience OR capacity OR competenc^∗^) AND
 (pandemic^∗^ OR epidemic^∗^ OR disaster^∗^ OR emergenc^∗^ OR outbreak^∗^)

**TABLE 1:** Search string used for database search.

Search component (TITLE‐ABS‐KEY)	Justification
nurse^∗^ OR “nursing workforce” OR “nursing profession”	Ensures comprehensive retrieval of literature explicitly related to nursing and the nursing workforce. The wildcard (^∗^) captures lexical variations (e.g., nurse, nurses, and nursing), while workforce‐ and profession‐specific terms broaden coverage to organizational, professional, and system‐level nursing studies.
preparedness OR readiness OR resilience OR capacity OR competenc^∗^	Captures key constructs used to conceptualize nursing workforce preparedness. These terms reflect complementary dimensions of preparedness, including operational readiness, adaptive capacity, professional competence, and psychological or system‐level resilience. The wildcard (^∗^) allows retrieval of related forms (e.g., competency and competencies).
pandemic^∗^ OR epidemic^∗^ OR disaster^∗^ OR emergenc^∗^ OR outbreak^∗^	Defines the health emergency context in which nursing workforce preparedness is examined. The use of broad emergency‐related terms ensures inclusion of studies across diverse hazards, including infectious disease outbreaks, large‐scale disasters, and public health emergencies. The wildcard (^∗^) captures singular and plural variants, including emergency and emergencies through the term emergenc^∗^.

Scopus was selected as the sole bibliographic database because the study aimed to generate a consistent science‐mapping corpus with standardized citation, affiliation, author keyword, index keyword, source, and cited‐reference metadata. The use of a single database reduced cross‐database duplication, indexing inconsistencies, and metadata harmonization issues that may occur when merging records from Scopus, Web of Science, and PubMed. Web of Science was not combined with Scopus because cross‐database merging would require additional normalization of cited references, author names, source titles, and institutional affiliations, which may introduce inconsistencies into co‐citation and co‐word networks. PubMed was not used as a parallel source because it is primarily biomedical and does not consistently provide the same citation and cited‐reference metadata needed for co‐citation mapping. Thus, Scopus was used to maintain analytic consistency and reproducibility across co‐citation, co‐word, thematic evolution, hotspot, and collaboration analyses. The implications of this single‐database approach are acknowledged in the Limitations and Future Works section.

Query validation was conducted through conceptual boundary checking and post‐export metadata auditing. The three concept blocks were designed to ensure that each retrieved record contained a nursing‐related concept, a preparedness/resilience‐related concept, and a health emergency context. The search terms were selected to capture lexical variants used in the literature, including nurse/nurses/nursing, preparedness/readiness/resilience/capacity/competence, and pandemic/epidemic/disaster/emergency/outbreak. After export, the dataset was further checked using titles, abstracts, author keywords, and index keywords to determine whether records remained conceptually aligned with the study focus.

The raw Scopus export yielded 9010 records and contained publication years from 1941 to 2026. Records were then filtered according to the predefined study scope of 2000–2025, English‐language publication, and eligible document types. Full bibliographic records were exported in CSV format, including titles, abstracts, author keywords, index keywords, cited references, citation counts, source titles, affiliations, and document‐type information. The complete study identification, filtering, metadata‐based screening, and corpus inclusion process is shown in Supporting Figure 1.

### 2.3. Eligibility Criteria

Records were eligible for inclusion if they were indexed in Scopus, published between 2000 and 2025, written in English, and classified as peer‐reviewed journal articles or review articles. Editorials, letters, commentaries, notes, conference papers, book chapters, books, short surveys, errata, conference reviews, retracted items, and data papers were excluded because they were not part of the target peer‐reviewed article and review corpus.

The screening workflow was conducted in sequential stages. First, the raw Scopus export contained 9010 records. After limiting records to the target period of 2000–2025, 8644 records remained. The English‐language filter excluded 414 non‐English records, leaving 8230 records. Document‐type filtering retained only articles and reviews, resulting in 7650 records. Duplicate records were then checked using normalized title information, which removed 19 duplicate titles and left 7631 records for metadata‐based content screening.

Because broad bibliometric search strings may retrieve records that are technically captured by the search query but only weakly aligned with the intended conceptual scope, an additional metadata‐based scope audit was conducted. Titles, abstracts, author keywords, and index keywords were examined to ensure that retained records contained all three required conceptual elements: at least one nursing‐related term, at least one preparedness/resilience‐related term, and at least one emergency‐context term. This step excluded 61 records that contained nursing and emergency‐context terms but did not contain a preparedness, readiness, resilience, capacity, or competence term. The final retained corpus included 7570 documents, composed of 6950 articles and 620 reviews. This corpus was used for the bibliometric analysis and VOSviewer mapping.

### 2.4. Data Cleaning and Keyword Harmonization

Data cleaning was performed before bibliometric mapping. Duplicate records were identified using normalized title information, in which titles were converted to lowercase and stripped of excess spacing to detect repeated entries. Metadata fields were inspected for completeness, particularly titles, abstracts, author keywords, index keywords, cited references, publication year, document type, and language.

Keyword harmonization was conducted to improve conceptual consistency during co‐word and thematic analyses. Similar terms, spelling variants, singular/plural forms, and closely related expressions were checked and standardized where appropriate. Examples included terms related to COVID‐19, coronavirus disease 2019, SARS‐CoV‐2, pandemic, pandemics, disaster preparedness, emergency preparedness, nursing workforce, nurses, and nursing staff. An additional thesaurus‐based keyword‐cleaning step was conducted for the co‐word analysis. Generic Scopus indexing terms and broad demographic descriptors, including human, humans, female, male, adult, aged, middle aged, young adult, article, priority journal, controlled study, major clinical study, human experiment, procedures, physician, and related nonconceptual descriptors, were excluded from the final interpreted co‐word visualization. Methodological descriptors, including questionnaire, questionnaires, surveys and questionnaires, cross‐sectional study/studies, qualitative research, interview, review, methodology, standard, standards, and practice guideline, were excluded from the substantive co‐word interpretation. Synonymous or near‐equivalent variants were consolidated or interpreted under common conceptual domains, including nurse/nurses/nursing as nursing/nurses, COVID‐19/coronavirus disease 2019/SARS‐CoV‐2 as COVID‐19, pandemic/pandemics as pandemic(s), health personnel/healthcare personnel as healthcare personnel, and psychological resilience/resilience‐related variants as resilience. This additional cleaning reduced indexing noise and improved the conceptual accuracy and readability of the co‐word network. The full keyword harmonization and exclusion audit is provided in Supporting Table S8.

### 2.5. Network Parameterization and Thresholds

Network construction and visualization were performed using VOSviewer Version 1.6.20 with association‐strength normalization. Thresholds and clustering settings were selected to balance coverage, readability, connectedness, and conceptual interpretability. Lower thresholds were tested but produced denser maps with more weakly connected items, while higher thresholds removed potentially important references or keywords and fragmented the cluster structure. Final parameters were therefore selected based on whether the resulting networks retained sufficient coverage while producing coherent and interpretable clusters.

For co‐citation analysis, the retained corpus generated 69,391 cited reference entries and 50,287 unique cited references. In VOSviewer, 47,928 cited references were available for co‐citation counting. Three citation thresholds were examined. A threshold of 18 citations retained 72 cited references and produced a four‐cluster solution, but the network remained relatively broad and less focused. A threshold of 20 citations retained only 58 cited references and produced a six‐cluster solution, which was more fragmented and less conceptually stable. Therefore, a minimum citation threshold of 19 citations per cited reference was selected because it retained 65 cited references, produced a coherent four‐cluster solution, and provided the best balance between coverage and interpretability. After excluding one disconnected item, the final connected co‐citation map contained 64 cited references organized into four clusters. Cluster interpretation was based on citation weight, total link strength, and conceptual coherence among highly connected references.

After the VOSviewer co‐citation network was generated, a secondary cited‐reference relevance screening was conducted to address the distinction between network prominence and topical relevance. All 64 connected co‐cited references were reviewed and classified according to their role in the network: (1) directly aligned references focusing on nursing preparedness, disaster nursing competence, emergency workforce readiness, resilience, staffing, safety, or nursing workforce support; (2) methodological or measurement references, such as general qualitative analysis or resilience‐scale papers; and (3) context‐setting references, such as early COVID‐19 clinical and epidemiologic papers. Directly aligned references were prioritized in the main co‐citation table and narrative interpretation. Methodological, measurement, and context‐setting references were retained in the overall network interpretation only when they helped explain how the field was conceptually or methodologically structured, but they were not treated as core nursing preparedness frameworks.

For co‐word analysis, author keywords and index keywords were examined to map the conceptual structure of the field. After keyword cleaning, thesaurus‐based harmonization, and exclusion of generic indexing, demographic, and methodological descriptors, VOSviewer identified 19,122 keyword terms available for co‐occurrence analysis. Three occurrence thresholds were examined to assess parameter robustness. A threshold of 264 occurrences retained 61 keywords and produced a four‐cluster solution with cluster sizes of 22, 15, 14, and 10 keywords, but it retained an additional lower‐prominence term and produced a less balanced cluster structure. A threshold of 266 occurrences retained 59 keywords and produced a four‐cluster solution with cluster sizes of 24, 15, 13, and 7 keywords, but it removed one relevant conceptual term. Therefore, a minimum occurrence threshold of 265 was selected because it retained 60 keywords and produced a coherent four‐cluster structure with cluster sizes of 25, 15, 13, and 7 keywords. Network construction used association‐strength normalization in VOSviewer Version 1.6.20, with a resolution parameter of 1.06 and a minimum cluster size of six. These parameters provided the best balance between conceptual coverage, readability, and cluster stability.

For thematic evolution and hotspot analyses, author keywords were used because they capture author‐defined conceptual emphases. Of the 7570 retained documents, 5948 contained analyzable author keywords, while 1622 records lacked author‐keyword metadata and were excluded only from author‐keyword‐based thematic evolution and hotspot analyses. The full retained corpus was used for descriptive analysis, co‐citation mapping, and co‐word structural mapping.

## 3. Results

### 3.1. Descriptive Analysis

Authorship patterns were strongly collaborative. The retained dataset comprised 33,656 unique author‐name entries, with a mean of 5.25 authors per paper and a median of 4 authors per paper. Only 9.1% of publications were single‐authored, indicating that scholarship in this area is largely team‐based. Author productivity was concentrated among a small group of recurring contributors, with the most productive authors including Julie Considine, Tener Goodwin Veenema, Lisa Adams Wolf, Veronica Lindström, Leodoro J. Labrague, Terri L. Rebmann, and Simon Cooper Jr. These authors represent influential contributors across emergency nursing, disaster preparedness, resilience, and workforce‐related scholarship. Full author productivity indicators are presented in Supporting Table S6.

Institutional contributions were similarly concentrated in universities, schools of nursing, medical schools, hospitals, and public health institutions with sustained output in emergency nursing, disaster preparedness, workforce resilience, and health systems research. The most productive affiliation organizations included Monash University, University of California, Karolinska Institutet, University of Toronto, Johns Hopkins School of Nursing, Harvard Medical School, Deakin University, Johns Hopkins University School of Medicine, the University of British Columbia, and University of Pennsylvania. Full institutional contribution indicators are presented in Supporting Table S7.

**FIGURE 1:**
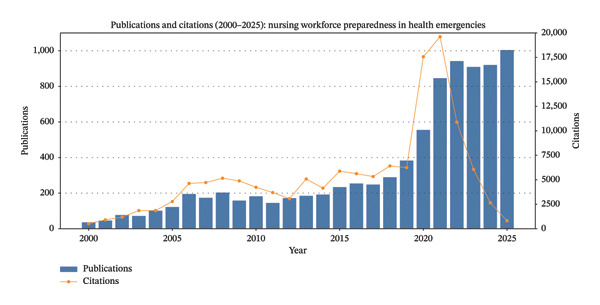
Annual publication and citation trends in nursing workforce preparedness research during health emergencies (2000–2025).

At the country level, 18.7% of unique papers with extractable affiliation data involved international or multicountry collaboration, while most outputs remained single‐country publications. The strongest international collaboration links included Canada–United States, Australia–United Kingdom, United Kingdom–United States, and Australia–United States. Geographic distribution was global but concentrated in a limited number of high‐producing countries and collaboration hubs. Supporting Tables S2 and S3 report country collaboration links and country‐attributed publication counts; in Supporting Table S3, multicountry papers were counted once for each participating country, so the country‐attributed denominator exceeds the number of retained documents. Annual publication and citation trends in nursing workforce preparedness research during health emergencies from 2000 to 2025 are presented in Figure 1.

### 3.2. Co‐Citation Analysis

Co‐citation analysis was conducted to identify the intellectual foundations and conceptual anchors of research on nursing workforce preparedness and resilience in health emergencies. By examining how frequently cited references are cited together across the retained corpus, this method reveals the theoretical, empirical, methodological, and context‐setting works that have most strongly shaped the field. Because co‐citation prominence does not automatically indicate direct topical alignment, the co‐cited references were interpreted according to their role in the network rather than treated uniformly as direct nursing workforce preparedness frameworks. From the final retained dataset, VOSviewer identified 47,928 cited references. A minimum citation threshold of 19 citations per cited reference was applied to focus the analysis on influential and sufficiently connected works. Although 65 cited references met this threshold, the final connected co‐citation map exported from VOSviewer comprised 64 cited references, organized into four clusters. After the VOSviewer network was generated, a secondary cited‐reference relevance screening was conducted. The 64 connected co‐cited references were classified as directly aligned references, methodological or measurement anchors, or context‐setting references. Directly aligned references were prioritized in the main co‐citation table and core interpretation, while methodological, measurement, and context‐setting references were retained only to explain the broader intellectual and contextual structure of the field.

The network also contained broader contextual and methodological anchors, including pandemic‐era nursing education and professional‐experience sources [37, 38], a scoping‐review framework and statistical‐power guidance [39, 40], self‐efficacy theory [41], nursing education and professional‐formation scholarship [42], a pediatric emergency‐service readiness assessment [43], and diagnostic‐classification guidance relevant to mental‐health research [44]. These references were used to interpret the intellectual structure of the co‐citation network and were not treated as direct evidence of nursing workforce preparedness.

The resulting network shows that nursing workforce preparedness scholarship is structured around four interrelated intellectual foundations: (1) psychological resilience and mental health during health emergencies; (2) disaster nursing preparedness, competence, and willingness to respond; (3) workforce structures, professional formation, staffing, and safety; and (4) COVID‐19 hazard‐context evidence and frontline risk. This four‐cluster structure indicates that the field has moved beyond a narrow disaster‐competency focus and now integrates psychological sustainability, workforce systems, professional development, and pandemic‐specific evidence.

At the center of the network are disaster nursing preparedness and competence studies, led by highly connected works such as Baack and Alfred, Al Thobaity et al., and Al Khalaileh et al.

After secondary cited‐reference relevance screening, the strongest directly aligned co‐cited references were concentrated in disaster nursing preparedness, nursing competence, emergency readiness, and nursing workforce response. Highly connected works by Baack and Alfred, Al Thobaity et al., Al Khalaileh et al., Al Harthi et al., Chegini et al., Labrague et al., Chapman and Arbon, and Fung et al. formed the field‐specific empirical backbone of the network. These references form the empirical backbone of the field, showing that perceived preparedness, disaster competence, readiness, and willingness to respond remain enduring anchors in nursing emergency scholarship. They also demonstrate that knowledge, skills, disaster nursing education, and emergency‐response capacity remain central foundations of nursing workforce preparedness in health emergencies. Their high total link strength indicates that competency assessment and preparedness measurement continue to connect studies across different hazards, countries, and practice settings (see Table 2).

**TABLE 2:** Top 10 directly aligned co‐cited references in nursing workforce preparedness and resilience research.

No	Documents	Citation	Total link strength
1	Baack S, Alfred D. Nurses’ preparedness and perceived competence in managing disasters. Journal of Nursing Scholarship. 2013; 45 (3): 281–287. https://doi.org/10.1111/jnu.12029	71	86
2	Al Thobaity A, Plummer V, Williams B. What are the most common domains of the core competencies of disaster nursing? A scoping review. International Emergency Nursing. 2017; 31: 64–71. https://doi.org/10.1016/j.ienj.2016.10.003	43	62
3	Al Thobaity A, Plummer V, Innes K, Copnell B. Perceptions of knowledge of disaster management among military and civilian nurses in Saudi Arabia. Australasian Emergency Nursing Journal. 2015; 18 (3): 156–164. https://doi.org/10.1016/j.aenj.2015.03.001	34	57
4	Al Khalaileh MA, Bond E, Alasad JA. Jordanian nurses’ perceptions of their preparedness for disaster management. International Emergency Nursing. 2012; 20 (1): 14–23. https://doi.org/10.1016/j.ienj.2011.01.001	37	56
5	Al Harthi M, Al Thobaity A, Al Ahmari W, Almalki M. Challenges for nurses in disaster management: A scoping review. Risk Management and Healthcare Policy. 2020; 13: 2627–2634. https://doi.org/10.2147/RMHP.S279513	42	42
6	Chegini Z, Arab‐Zozani M, Kakemam E, Lotfi M, Nobakht A, Aziz Karkan H. Disaster preparedness and core competencies among emergency nurses: A cross‐sectional study. Nursing Open. 2022; 9 (2): 1294–1302. https://doi.org/10.1002/nop2.1172	26	40
7	Achora, S., & Kamanyire, J. K. (2016). Disaster Preparedness: Need for inclusion in undergraduate nursing education. Sultan Qaboos University Medical Journal, 16 (1), e15–e19. https://doi.org/10.18295/squmj.2016.16.01.004	19	36
8	Labrague LJ, Hammad K, Gloe DS, McEnroe‐Petitte DM, Fronda DC, Obeidat AA, et al. Disaster preparedness among nurses: a systematic review. Int Nurs Rev. 2018; 65 (1): 41–53. https://doi.org/10.1111/inr.12369	37	34
9	Fung OW, Loke AY, Lai CK. Disaster preparedness among Hong Kong nurses. J Adv Nurs. 2008; 62 (6): 698–703. https://doi.org/10.1111/j.1365-2648.2008.04655.x	33	33
10	Chapman K, Arbon P. Are nurses ready? Australas Emerg Nurs J. 2008; 11 (3): 135–44. https://doi.org/10.1016/j.aenj.2008.04.002	29	33

*Note:* References in this table were selected from the connected VOSviewer co‐citation network after secondary cited‐reference relevance screening. Only references directly aligned with nursing preparedness, disaster nursing competence, emergency nursing readiness, nursing education for disaster response, or nursing workforce resilience were included in the main table. The table is ranked by total link strength among directly aligned references; when total link strength was equal, citation count was used as the secondary ordering criterion. Highly co‐cited but indirectly aligned references, such as general resilience scales, qualitative methodology papers, and early COVID‐19 clinical or epidemiologic reports, were retained in the broader network interpretation as measurement, methodological, or context‐setting anchors, but they were not treated as core nursing preparedness frameworks.

A second major intellectual strand concerns psychological resilience and mental health during health emergencies. Highly co‐cited references such as Lai et al., Connor and Davidson, Pappa et al., and Adams and Walls demonstrate how pandemic‐era scholarship expanded preparedness discourse from technical readiness toward emotional endurance, stress adaptation, burnout prevention, and workforce sustainability. The inclusion of methodological anchors such as Braun and Clarke also indicates the field’s reliance on qualitative and mixed‐methods approaches to understand lived experiences, coping, and organizational strain during crises. Several highly co‐cited references were retained as supporting anchors rather than core field‐specific foundations. For example, Connor and Davidson functioned as a resilience‐measurement reference, Braun and Clarke as a qualitative‐methodology reference, and early COVID‐19 clinical and epidemiologic studies as hazard‐context references. These works were not interpreted as direct nursing preparedness frameworks; instead, they were used to explain how nursing workforce preparedness research drew on broader resilience measurement, qualitative inquiry, and pandemic‐context evidence.

The third cluster highlights workforce structure and professional formation. This cluster includes foundational works on staffing, education, professional competence, patient safety, and the development of nursing expertise, including works associated with Aiken, Benner, professional nursing education standards, and patient safety scholarship. This suggests that preparedness is not only an individual competency but also a system‐level capability shaped by staffing adequacy, education, safety culture, and organizational infrastructure.

The fourth cluster reflects COVID‐19 hazard‐context evidence. References such as Huang et al., Wang et al., Guan et al., Zhu et al., Fernandez et al., and WHO pandemic‐related sources were frequently co‐cited to establish the clinical, epidemiologic, and occupational risk context of COVID‐19. These references function primarily as context‐setting works rather than as foundational nursing preparedness theories. Their presence indicates that pandemic‐era nursing workforce research drew heavily on early COVID‐19 evidence to frame uncertainty, frontline risk, workforce exposure, and the need for rapid system adaptation.

Overall, the updated co‐citation structure demonstrates that nursing workforce preparedness and resilience research is a practice‐driven and system‐oriented field. Its intellectual base integrates disaster nursing competence, psychological resilience, professional formation, staffing and safety, and pandemic‐context evidence. The revised interpretation distinguishes between directly aligned nursing workforce preparedness references and broader methodological, measurement, or hazard‐context references, thereby improving the conceptual accuracy of the co‐citation findings. This supports the study’s broader argument that preparedness should be understood as a multidimensional workforce strategy rather than a narrow technical or disaster‐planning function.

The secondary screening clarified the role of several highly co‐cited but indirectly aligned references. Connor and Davidson’s resilience‐scale paper was classified as a measurement anchor because it informed how resilience is operationalized across health workforce studies. Braun and Clarke’s thematic analysis paper was classified as a methodological anchor because it supported qualitative inquiry on lived experiences, coping, and organizational strain. Early COVID‐19 clinical and epidemiologic papers were classified as context‐setting anchors because they established the hazard environment that shaped nursing workforce risk, preparedness, and adaptation. These references were therefore retained for interpretive completeness but were not presented as direct nursing workforce preparedness studies.

Co‐citation analysis further identified four interrelated thematic clusters that define the intellectual structure of nursing workforce preparedness and resilience research in health emergencies. Each cluster represents a body of literature frequently cited together, reflecting shared conceptual, empirical, methodological, or context‐setting orientations. Total link strength highlights references that function as conceptual anchors connecting research on preparedness, resilience, competence, mental health, staffing, safety, and workforce capacity.

**FIGURE 2:**
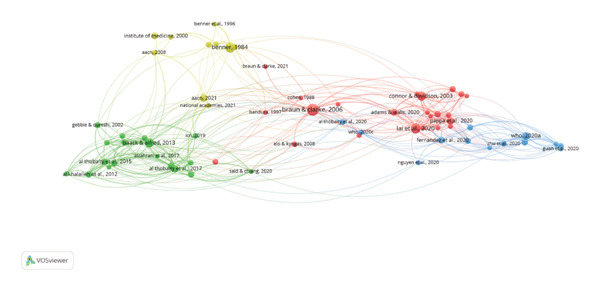
Co‐citation network of key literature on nursing workforce preparedness in health emergencies.

The co‐citation map in Figure 2 illustrates four clusters: psychological resilience and mental health during health emergencies; disaster nursing preparedness and core competence; workforce structures, professional formation, staffing, and safety; and COVID‐19 hazard‐context evidence. Strong linkages between pre‐pandemic disaster nursing studies and pandemic‐era workforce research indicate intellectual continuity, suggesting that COVID‐19 expanded rather than replaced earlier preparedness frameworks.

• Cluster 1 (green): *“Psychological preparedness, resilience, and mental health protection in health emergencies.”* This cluster includes 23 cited references/items and represents the psychosocial foundation of workforce preparedness. Highly co‐cited studies on COVID‐19 mental health outcomes, burnout, trauma exposure, and resilience measurement show that preparedness is increasingly framed as a human‐capacity issue, not only a technical or logistical function [2–6, 8, 14–16, 45, 46]. The presence of qualitative and methodological references also indicates the importance of lived‐experience research in understanding stress, coping, and organizational support during emergencies [47].
• Cluster 2 (red): “*Disaster nursing preparedness, core competence, willingness, and leadership.*” This cluster includes 18 cited references/items and forms the practice‐facing foundation of the field. The highly connected works of Baack and Alfred, Al Thobaity et al., Al Khalaileh et al., and related disaster nursing studies show that preparedness is commonly measured through perceived competence, knowledge, skills, and readiness to respond [20, 30–32, 48]. The cluster also links preparedness with willingness to report to duty, competency frameworks, and leadership expectations, suggesting that surge capacity depends on both individual capability and organizational support [9–11, 19, 21–23, 25, 26, 49].
• Cluster 3 (blue): *“COVID-19 hazard-context evidence, frontline risk, and acute-care experience.”* This cluster includes 13 cited references/items and reflects the pandemic‐context evidence used to frame risk, uncertainty, and rapid system adaptation. Early COVID‐19 clinical and epidemiologic studies, frontline risk evidence, and qualitative accounts of healthcare workers’ experiences were frequently co‐cited to establish the hazard environment rather than to provide nursing preparedness theory [50–57]. This cluster therefore functions as a context‐setting layer that explains why workforce protection, mental health, and adaptive response became more prominent after 2020.
• Cluster 4 (yellow): “*Workforce structures, professional formation, staffing, safety, and education standards.”* This cluster includes 10 cited references/items and highlights the structural conditions that enable preparedness. Staffing, safety, education, and professional‐development references indicate that preparedness is not only an individual attribute but also a system‐level workforce capability [12, 13, 17, 18, 58–61]. The cluster supports the interpretation that emergency readiness depends on staffing adequacy, safety culture, education standards, and progressive competence development.

Taken together, the four‐cluster co‐citation structure shows that nursing workforce preparedness research is a multidimensional field integrating disaster competence, psychosocial resilience, workforce systems, and pandemic‐context evidence. The map suggests that COVID‐19 expanded earlier disaster preparedness frameworks by making mental health protection, staffing, safety, and system readiness more central to nursing preparedness scholarship (see Table 3).

**TABLE 3:** Co‐citation clusters underpinning nursing workforce preparedness and resilience research in health emergencies.

Cluster	Cluster label	Number of cited references/items	Representative publications
1 (green)	Psychological preparedness, resilience, and mental health protection	23	Lai et al., 2020; Connor & Davidson, 2003; Braun & Clarke, 2006; Adams & Walls, 2020; Pappa et al., 2020; Chen et al., 2020; Campbell‐Sills & Stein, 2007; Chew et al., 2020; Cooper et al., 2020; Brooks et al., 2020; Galanis et al., 2021; Baskin & Bartlett, 2021; Dewart et al., 2020; Jackson et al., 2020
2 (red)	Disaster nursing preparedness, core competence, willingness, and leadership	18	Baack & Alfred, 2013; Al Thobaity et al., 2017; Al Thobaity et al., 2015; Al Khalaileh et al., 2012; Al Harthi et al., 2020; Chegini et al., 2022; Labrague et al., 2018; Chapman & Arbon, 2008; Fung et al., 2008; Veenema et al., 2016; ICN, 2009; ICN, 2019; Arbon et al., 2013; Said & Chiang, 2020; Gebbie & Qureshi, 2002; Alzahrani et al., 2017
3 (blue)	COVID‐19 hazard‐context evidence, frontline risk, and acute‐care experience	13	Huang et al., 2020; Wang et al., 2020; Guan et al., 2020; Wu & McGoogan, 2020; Zhu et al., 2020; Nguyen et al., 2020; Liu et al., 2020; Fernandez et al., 2020; Cucinotta & Vanelli, 2020; WHO, 2020a–d
4 (yellow)	Workforce structures, professional formation, staffing, safety, and education standards	10	Benner, 1982/1984; Benner et al., 1996; Benner et al., 2010; Aiken et al., 2002; Aiken et al., 2014; AACN, 2008; AACN, 2021; National Academies, 2021; Institute of Medicine, 2000

*Note:* Cluster sizes refer to cited references/items in the VOSviewer co‐citation network, not necessarily journal articles. The network includes empirical studies, reviews, books, professional standards, reports, and methodological references. Representative references were selected based on citation weight, total link strength, and conceptual relevance within each cluster. Cited‐reference node labels were standardized to a short author/year or organization/year format for readability. Label standardization did not alter citation counts, total link strength, cluster assignment, node position, or the underlying co‐citation network structure.

### 3.3. Co‐Word Analysis

Co‐word analysis was conducted to map the conceptual structure of nursing workforce preparedness and resilience research in health emergencies. Using the retained corpus of 7570 documents, VOSviewer identified 19,122 keyword terms available for co‐occurrence analysis after keyword cleaning and thesaurus‐based harmonization. To address indexing noise and improve conceptual accuracy, generic demographic, database‐indexing, and methodological descriptors were excluded from the substantive interpretation. A minimum occurrence threshold of 265 was applied, yielding 60 high‐frequency keywords for the harmonized co‐word network. Network construction used association‐strength normalization in VOSviewer Version 1.6.20, with a resolution parameter of 1.06 and a minimum cluster size of six. The final network produced a coherent four‐cluster solution composed of 25, 15, 13, and 7 keywords.

For interpretive clarity, generic indexing terms and broad demographic descriptors such as human, humans, female, male, adult, aged, middle aged, young adult, article, priority journal, controlled study, major clinical study, human experiment, procedures, physician, and related nonconceptual descriptors were excluded from the final interpreted co‐word visualization. Additional thesaurus‐based harmonization was conducted for high‐frequency lexical variants, including nurse/nurses/nursing as nursing/nurses, COVID‐19/coronavirus disease 2019/SARS‐CoV‐2 as COVID‐19, pandemic/pandemics as pandemic(s), health personnel/healthcare personnel as healthcare personnel, and psychological resilience/resilience‐related variants as resilience. Methodological descriptors, including questionnaire, questionnaires, surveys and questionnaires, cross‐sectional study/studies, qualitative research, interview, review, methodology, standard, standards, and practice guideline, were not treated as substantive conceptual domains. The harmonization and exclusion decisions are documented in Supporting Table S8.

Across the four clusters, the harmonized co‐word network shows that nursing workforce preparedness is conceptualized as a multidimensional construct integrating clinical competence and acute‐care delivery, pandemic‐related psychological burden and resilience, workforce organization and emergency service capacity, and nursing education for disaster preparedness. The harmonized network indicates that preparedness scholarship is not limited to disaster response but extends to clinical competence, emergency care delivery, nursing education, organizational management, leadership, staffing capacity, mental health protection, resilience, and service continuity.

**FIGURE 3:**
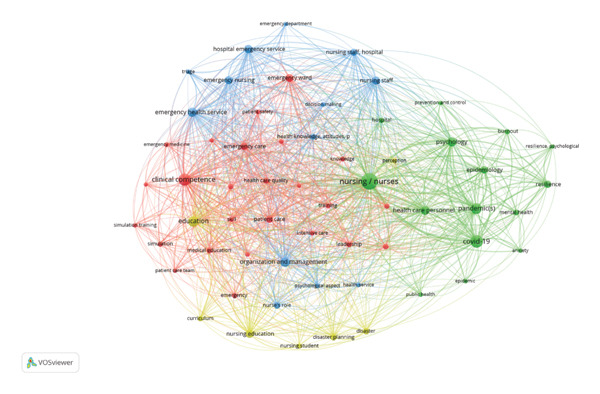
Harmonized co‐word network of core themes in nursing workforce preparedness for health emergencies.

Table 4 presents the top harmonized substantive concepts in the co‐occurrence analysis, while Figure 3 illustrates the harmonized co‐word network.

**TABLE 4:** Top 15 harmonized conceptual terms in the co‐occurrence analysis.

Ranking	Keyword	Occurrences	Total link strength
1	nursing/nurses	3602	18,015
2	clinical competence	1952	11,561
3	COVID‐19	2097	9383
4	education	1441	9128
5	pandemic(s)	1569	7841
6	organization and management	1207	7735
7	psychology	1135	6892
8	emergency health service	1104	6359
9	nursing staff	871	6049
10	healthcare personnel	1030	5926
11	hospital emergency service	901	5469
12	nursing education	901	5322
13	emergency nursing	848	5176
14	nursing staff, hospital	673	4969
15	emergency ward	847	4914

*Note:* Generic demographic, database‐indexing, and methodological descriptors were excluded from the final substantive co‐word table to improve conceptual clarity. Closely related variants were harmonized before final interpretation: nurse/nurses/nursing were grouped as nursing/nurses; COVID‐19/coronavirus disease 2019/SARS‐CoV‐2 as COVID‐19; pandemic/pandemics as pandemic(s); health personnel/healthcare personnel as healthcare personnel; and psychological resilience/resilience‐related variants were interpreted within the resilience domain. Methodological terms, including questionnaire, questionnaires, surveys and questionnaires, cross‐sectional study/studies, qualitative research, interview, review, methodology, standard, standards, and practice guideline, were not treated as substantive conceptual domains.

Viewed collectively, the harmonized co‐word network in Figure 3 reveals four interrelated conceptual domains: clinical competence, emergency and acute‐care delivery, and training; COVID‐19, psychological burden, resilience, and health workforce risk; workforce organization, emergency service capacity, and nursing staff roles; and nursing education and disaster preparedness. Methodological descriptors that appeared prominently in the preliminary export were excluded from the substantive table and conceptual interpretation to prevent study‐design or instrument terms from being interpreted as preparedness domains. The network shows that preparedness scholarship is anchored in clinical competence, education, emergency nursing, disaster planning, organization and management, leadership, COVID‐19, psychological resilience, mental health, nursing workforce capacity, and emergency care delivery. Although explicitly future‐oriented terms remain limited, the co‐occurrence of competence, resilience, leadership, education, staffing, and system‐level concepts suggests that nursing scholarship increasingly addresses future readiness implicitly through preparedness, capacity building, adaptive workforce design, and service continuity.

• Cluster 1 (red): *“Clinical competence, emergency and acute-care delivery, and training.”* This domain includes 25 keywords and is represented by terms such as clinical competence, emergency ward, patient care, emergency care, healthcare quality, leadership, medical education, healthcare delivery, training, skill, simulation, intensive care unit, emergency, knowledge, patient care team, intensive care, emergency medicine, nurse practitioner, interpersonal communication, clinical practice, patient safety, resuscitation, total quality management, professional competence, and simulation training. These terms indicate that preparedness is strongly grounded in professional competence, service delivery, clinical practice, skills development, leadership, patient safety, and care quality in high‐pressure emergency and acute‐care environments. This aligns with evidence emphasizing emergency nursing competencies, simulation‐based training, leadership development, and safe care delivery as essential components of workforce readiness during crises [9–11, 13, 20, 23, 25, 30–32, 48].
• Cluster 2 (green): *“COVID-19, psychological burden, resilience, and health workforce risk.”* This domain includes 15 keywords and is represented by nursing/nurses, COVID‐19, pandemic(s), psychology, healthcare personnel, epidemiology, resilience, mental health, hospital, burnout, resilience, psychological, anxiety, public health, prevention and control, and epidemic. These terms show that pandemic‐era scholarship shifted preparedness research toward workforce well‐being, psychological risk, infection‐related uncertainty, adaptive capacity, prevention and control, and mental health protection. This cluster reflects evidence documenting stress, burnout, anxiety, and psychological distress among nurses and healthcare workers during the COVID‐19 pandemic, while highlighting resilience as a critical protective factor for sustaining workforce performance and retention [4, 5, 7, 8, 14–16, 27, 45, 56, 57].
• Cluster 3 (blue): “Workforce organization, emergency service capacity, and nursing staff roles.” This domain includes 13 keywords and is represented by organization and management, emergency health service, nursing staff, hospital emergency service, emergency nursing, nursing staff, hospital, nurse’s role, health knowledge, attitudes, practice, psychological aspect, triage, health service, decision making, and emergency department (ED). These terms show that preparedness is commonly conceptualized as a structured workforce and organizational capability supported by emergency nursing practice, role clarity, staffing, triage, decision‐making, and system‐level management. The prominence of these concepts supports the interpretation that workforce preparedness depends not only on individual competencies but also on organizational support, staffing adequacy, leadership, and coordinated emergency response systems [17, 18, 21–23, 30–32, 59].
• Cluster 4 (yellow): *“Nursing education and disaster preparedness.”* This domain includes 7 keywords and is represented by education, nursing education, disaster planning, disaster, curriculum, nursing student, and perception. These terms indicate that disaster preparedness remains closely linked with nursing education, curriculum development, student preparation, and formal disaster planning. This supports prior work emphasizing the integration of disaster preparedness competencies into nursing curricula to ensure that future nurses possess the knowledge, skills, and confidence required for effective emergency response and disaster management [9–11, 13, 25, 26, 30–32, 48, 49].

Overall, the harmonized co‐word network shows that nursing workforce preparedness is organized around clinical competence and acute‐care delivery, pandemic‐related psychological burden and resilience, workforce organization and emergency service capacity, and nursing education for disaster preparedness. By excluding methodological descriptors from the substantive table and interpretation, the revised analysis more clearly distinguishes between what the field studies conceptually and how the field has commonly studied it. These findings support interpreting preparedness as a multilevel workforce strategy involving clinical capability, education, leadership, staffing capacity, mental health protection, emergency service continuity, and system readiness (see Table 5).

**TABLE 5:** Co‐word clusters and representative high‐link keywords in nursing workforce preparedness research.

Cluster no and color	Cluster label	Number of keywords	Representative keywords
1 (red)	Clinical competence, emergency and acute‐care delivery, and training	25	clinical competence; emergency ward; patient care; emergency care; healthcare quality; leadership; medical education; healthcare delivery; training; skill; simulation; intensive care unit; emergency; knowledge; patient care team; intensive care; emergency medicine; nurse practitioner; interpersonal communication; clinical practice; patient safety; resuscitation; total quality management; professional competence; simulation training
2 (green)	COVID‐19, psychological burden, resilience, and health workforce risk	15	nursing/nurses; COVID‐19; pandemic(s); psychology; healthcare personnel; epidemiology; resilience; mental health; hospital; burnout; resilience, psychological; anxiety; public health; prevention and control; epidemic
3 (blue)	Workforce organization, emergency service capacity, and nursing staff roles	13	organization and management; emergency health service; nursing staff; hospital emergency service; emergency nursing; nursing staff, hospital; nurse’s role; health knowledge, attitudes, practice; psychological aspect; triage; health service; decision making; emergency department
4 (yellow)	Nursing education and disaster preparedness	7	education; nursing education; disaster planning; disaster; curriculum; nursing student; perception

*Note:* Cluster sizes refer to keywords included in the harmonized VOSviewer co‐word network at the minimum occurrence threshold of 265. Generic demographic, database‐indexing, and methodological descriptors were excluded from the substantive interpretation to reduce indexing noise and improve conceptual accuracy. Representative keywords were selected based on occurrence, total link strength, and conceptual relevance within each cluster.

## 4. Discussion

### 4.1. Thematic Evolution Across Time

To explicitly examine thematic evolution, the final retained corpus was time‐sliced into three periods reflecting early development, expansion, and pandemic‐era acceleration: 2000–2009 (*n* = 981), 2010–2019 (*n* = 1989), and 2020–2025 (*n* = 4600). As thematic evolution and hotspot detection aimed to capture author‐generated conceptual framing, these analyses were conducted using author keywords. Of the 7570 retained documents, 5948 contained analyzable author‐keyword data, while 1622 records lacked author‐keyword metadata and were excluded only from author‐keyword‐based thematic evolution and hotspot analyses. The full retained corpus was used for the main bibliometric analyses, including descriptive analysis, co‐citation mapping, and co‐word structural mapping.

Comparison of author‐keyword profiles across periods reveals a clear shift in the conceptual orientation of nursing preparedness and resilience research in health emergencies. During 2000–2009, the literature was primarily operational, emphasizing emergency nursing practice and ED functions, with frequent terms such as emergency nursing, ED, triage, and resuscitation reflecting acute response priorities. Between 2010 and 2019, the field expanded toward preparedness training and system readiness, evidenced by increased prominence of simulation, disaster nursing, resilience, leadership, and burnout, alongside continued focus on ED and triage‐related concepts. In 2020–2025, the thematic center shifted markedly toward pandemic‐driven workforce well‐being and coping. This period was dominated by COVID‐19 and closely linked constructs including resilience, burnout, mental health, anxiety, depression, stress, and telemedicine, consistent with the post‐2020 publication surge. Collectively, these patterns indicate a progression from an operational emergency‐response literature to a more multidimensional preparedness agenda integrating professional competence, psychosocial sustainability, and system adaptation under prolonged crisis conditions. A periodized summary of top keywords is provided in Supporting Table S4.

### 4.2. Emerging Hotspots

Emerging hotspots were identified through differential author‐keyword growth comparing the 2020–2025 and 2010–2019 periods, supplemented by a recency indicator based on average publication year. Author keywords were normalized, counted once per paper, and compared across periods to identify topics with marked post‐2020 growth. The strongest emerging topics clustered around pandemic‐era workforce strain and psychological adaptation, indicating a rapid reorientation of preparedness scholarship toward sustaining the nursing workforce during prolonged emergencies. The most pronounced growth was observed for COVID‐19 (1155 vs. 0), resilience (391 vs. 29), pandemic (250 vs. 11), burnout (186 vs. 9), mental health (166 vs. 21), qualitative research (166 vs. 22), anxiety (96 vs. 5), stress (100 vs. 10), and depression (81 vs. 2). Technology‐enabled care terms also increased after 2020, although at lower absolute frequencies, including telehealth (37 vs. 3), telemedicine (30 vs. 8), and virtual care (7 vs. 0). Recency indicators further reinforced hotspot status, with COVID‐19 pandemic (APY = 2022.92), psychological resilience (APY = 2022.81), burnout and moral distress (APY = 2022.71), COVID‐19 (APY = 2022.48), and telehealth (APY = 2022.42) among the more recent terms. Together, these indicators show that post‐2020 scholarship increasingly frames workforce preparedness as inseparable from psychosocial sustainability, moral distress dynamics, resilience, and technology‐enabled service continuity. Ranked hotspot indicators are presented in Supporting Table S5.

### 4.3. Scientific Interpretation of Cluster Shifts and Post‐2020 Resilience Dominance

The cluster structure indicates that nursing workforce preparedness has evolved from a primarily competency‐ and response‐oriented field into a broader workforce sustainability and systems‐readiness agenda. The co‐citation clusters show that disaster nursing competence remains an enduring foundation, but it is now connected with psychological resilience, staffing, safety, leadership, and pandemic‐context evidence. Scientifically, this suggests that preparedness is no longer being conceptualized only as what nurses know or can do during emergencies; it is increasingly understood as the capacity of individuals, teams, and organizations to sustain safe care under prolonged disruption.

The dominance of resilience after 2020 can be interpreted as a response to the limits exposed by COVID‐19. Earlier preparedness literature emphasized disaster competence, emergency response roles, and willingness to report to duty, but the pandemic created a prolonged crisis in which technical preparedness alone was insufficient. The rapid growth of keywords such as resilience, burnout, mental health, moral distress, anxiety, depression, and stress indicates that the field increasingly recognized psychological endurance, recovery, and workforce protection as core preparedness concerns rather than secondary outcomes [4, 5, 8, 14–16, 27, 45, 46].

This shift also reveals blind spots in the earlier preparedness literature. First, preparedness was often measured through self‐reported competence, attitudes, and cross‐sectional surveys, leaving limited evidence on whether preparedness interventions improve workforce retention, care continuity, or patient safety during real emergencies. Second, technology‐enabled care, including telemedicine, telehealth, and virtual care, appears as an emerging but still underdeveloped area despite its relevance to service continuity during crises, as reflected in the post‐2020 hotspot pattern shown in Supporting Table S5. In this review, the rise of telemedicine‐related keywords suggests that digital care became part of the nursing preparedness agenda after 2020, particularly as health systems attempted to maintain care access while reducing infection exposure. Telemedicine can function as a service continuity tool by allowing selected consultations, follow‐up care, monitoring, education, and triage to continue when face‐to‐face services are disrupted [62, 63]. It can also support remote coordination by linking nurses, patients, families, and interprofessional teams during periods of isolation, quarantine, redeployment, or limited facility access [62, 63]. However, the hotspot pattern also suggests that telemedicine should not be viewed only as a technological solution. It may create new workforce demands, including digital communication workload, documentation burden, role adjustment, training needs, technology‐related stress, and unequal access to digital infrastructure [64]. Therefore, telemedicine preparedness should include nurse training, workflow redesign, digital competency development, leadership support, and evaluation of its effects on workload, care quality, and workforce well‐being [64]. Third, the field remains less developed in linking preparedness to organizational accountability, staffing policy, leadership readiness, and long‐term workforce recovery.

Taken together, the post‐2020 shift should be interpreted with caution. The dominance of COVID‐19 in the co‐citation, co‐word, and hotspot results may partly reflect pandemic‐era citation concentration, rapid publication growth, and the tendency of recent workforce studies to cite early COVID‐19 clinical, epidemiologic, and occupational‐risk evidence to justify crisis context. In this sense, some COVID‐19 prominence may represent a time‐bound surge rather than a stable intellectual foundation of nursing preparedness. However, the persistence of related themes such as resilience, burnout, moral distress, mental health, telemedicine, staffing, safety, and service continuity suggests that COVID‐19 also produced a more durable reorientation of the field (Table 4; Supporting Table S5). Rather than replacing earlier disaster preparedness frameworks, the pandemic expanded them by exposing weaknesses in event‐based preparedness systems that were less equipped for prolonged workforce strain [4, 5, 8, 27, 45, 46]. COVID‐19 therefore appears to function both as a citation‐amplifying shock event and as a turning point that pushed nursing workforce preparedness toward a broader system‐level agenda integrating competence, mental health protection, staffing adequacy, leadership, digital care continuity, and organizational support [17, 18, 23, 59, 62–64].

### 4.4. Implications of the Study

This bibliometric review provides a 25‐year map of nursing workforce preparedness and resilience research in health emergencies. For nursing managers, the findings indicate that preparedness should be operationalized as a measurable workforce strategy rather than treated as an episodic training requirement. The co‐citation, co‐word, thematic evolution, and hotspot findings suggest that nursing management practice should prioritize three linked areas: strengthening workforce policy and surge‐deployment systems, embedding resilience and mental health protection into emergency planning, and using organizational indicators to monitor workforce readiness and service continuity. These implications are particularly relevant for nurse executives, unit managers, workforce planners, and emergency preparedness committees responsible for maintaining safe care delivery during prolonged crises.

### 4.5. Leadership and Policy Recommendations

The mapped evidence supports a three‐pillar preparedness model for nursing management. These pillars are derived from the dominant bibliometric patterns: disaster competence and readiness in the co‐citation network, leadership and care delivery in the co‐word network, and post‐2020 hotspots related to resilience, burnout, moral distress, telemedicine, and mental health.

In practical terms, nursing managers can translate these findings into emergency‐ready workforce policies. These policies should define role‐specific emergency competencies, surge staffing thresholds, redeployment procedures, rest and recovery provisions, occupational safety requirements, and escalation pathways for staffing strain. Organizational capacity building should include regular simulation exercises, cross‐training for high‐demand units, leadership development for crisis decision‐making, and mechanisms for rapid communication between frontline nurses, managers, infection prevention teams, and executive leadership. Strategic preparedness planning should also include scenario‐based workforce plans for pandemics, disasters, outbreaks, and mass‐casualty events, with clear indicators for activation, staffing redistribution, staff protection, and post‐event recovery.

#### 4.5.1. Pillar 1: Competence and Readiness

Nurse managers and educators should integrate disaster and emergency preparedness competencies into undergraduate education, continuing professional development, simulation, drills, and role‐specific training. The bibliometric findings show that clinical competence, disaster planning, emergency nursing, and nursing education remain central concepts in the field [9–11, 13, 20, 30–32, 48]. Preparedness assessment should therefore include not only knowledge and attitudes but also observed performance, role clarity, and readiness to function during surge conditions. For implementation, managers may use annual competency mapping, role‐specific emergency drills, simulation‐based assessment, deployment‐readiness checklists, and staff orientation for emergency roles to identify training gaps before crises occur.

#### 4.5.2. Pillar 2: Resilience and Mental Health Protection

The post‐2020 growth of keywords such as burnout, mental health, moral distress, psychological resilience, and stress indicates that workforce well‐being has become a core preparedness concern. Nursing leaders should embed mental health support, burnout mitigation, peer support, debriefing, and moral distress support into emergency preparedness plans [2, 4–6, 8, 27, 45, 46]. These should be treated as preparedness measures rather than optional post‐crisis interventions. Operationally, this requires planned peer support, structured debriefing, fatigue management, rest‐period protection, moral distress support, and clear referral pathways for psychological care during and after emergencies.

#### 4.5.3. Pillar 3: System Supports and Service Continuity

The co‐word and co‐citation findings show that preparedness is also shaped by staffing, safety, leadership, healthcare quality, and organizational coordination. Nurse managers should strengthen surge staffing plans, safe work environments, risk communication, and leadership structures that protect both workforce well‐being and patient care continuity [17, 18, 23, 59]. Managers should therefore maintain surge staffing plans and workforce dashboards that monitor absenteeism, staffing strain, turnover risk, safety events, PPE or resource shortages, and service‐continuity indicators.

Overall, these implications translate the bibliometric findings into a practical nursing management agenda. Preparedness should not be limited to disaster training compliance; it should be planned, funded, monitored, and evaluated as a system‐level workforce capability.

### 4.6. Implications for Policy and Workforce Planning: Preparedness Is a Systems Capability

Across both co‐citation and co‐word analyses, preparedness is consistently framed as more than individual readiness. The prominence of system‐linked themes—staffing, safety, leadership, and organizational coordination—supports treating preparedness as a health‐system property that must be financed, governed, and monitored rather than addressed solely through training compliance [17, 24, 59]. Workforce readiness during emergencies is therefore strengthened when policy moves beyond episodic skills training toward structural protections, including staffing adequacy, safe work environments, clear role expectations, and surge‐capacity planning [18, 23]. In practice, this implies prioritizing scalable workforce protections and governance mechanisms that can be activated early in an emergency and sustained through prolonged disruption.

The post‐2020 inflection point further indicates that psychological risk is now central to preparedness agendas. Evidence demonstrating high levels of anxiety, depression, insomnia, and distress among healthcare workers during COVID‐19 confirms that workforce sustainability is inseparable from mental health protection [4, 5, 45]. Consequently, preparedness policies should explicitly embed psychological support as a core component of emergency readiness standards, reflecting both frontline realities and system accountability [2, 6]. From a policy perspective, preparedness thus becomes a composite investment in people, systems, and protection rather than a narrow compliance exercise. This shift supports including psychosocial safeguards (burnout mitigation and moral distress supports) as standard preparedness deliverables rather than optional add‐ons.

At the institutional level, nursing leaders should advocate for preparedness plans that are funded, rehearsed, and evaluated before emergencies occur. This includes protected time for training, budget allocation for workforce reserve capacity, policies for safe redeployment, and systems for monitoring staff well‐being and workforce availability. At the policy level, emergency preparedness standards should recognize nursing workforce readiness as a core component of health‐system resilience, alongside supplies, beds, equipment, and infection‐control infrastructure.

### 4.7. Implications for Nursing Education and Competency Development: Competence Frameworks Must Integrate Resilience

The strong linkage between competency‐centered disaster preparedness research and global standards suggests that education programs can use this field map to align curricula with established expectations while modernizing them for sustained and complex crises [9–11, 13]. The disaster preparedness literature consistently shows that perceived competence and readiness vary across contexts and that gaps persist even as training opportunities expand, making competency‐based education an ongoing priority [20, 25, 48]. Moving forward, education and training should more explicitly connect competency attainment to real‐world role performance during surge and redeployment conditions.

However, the intellectual shift toward psychological burden and resilience indicates that competence alone is insufficient for prolonged disruptions. Resilience is repeatedly operationalized and measured using validated tools [14, 15], and nursing‐specific conceptual work clarifies it as a professional capacity influencing performance, retention, and sustainability under adversity [16]. These findings suggest that education and continuing professional development should adopt an integrated preparedness model—clinical competence combined with psychological resilience and crisis leadership—rather than treating these domains as separate silos [13, 23]. Such integration better reflects the realities of extended health emergencies, where endurance, adaptability, and leadership are as critical as technical skill. Curricula and CPD can operationalize this integration through structured reflective debriefing, leadership‐in‐crisis competencies, and resilience‐informed supervisory practices.

### 4.8. Implications for Practice and Leadership: Readiness Is Demonstrated Through Care Continuity and Quality Under Pressure

The co‐word clusters show that preparedness is operationalized through emergency service delivery, patient care, procedures, and enabling leadership structures. This aligns with safety science perspectives, which emphasize that failures during crises often reflect system and process gaps rather than isolated individual shortcomings [59]. For nurse leaders, this implies that preparedness interventions should be evaluated not only by knowledge or attitude scores but by demonstrated service performance, safety continuity, and care quality during disruption, particularly in settings where staffing and work environments determine outcomes [17, 18]. Accordingly, leaders should pair preparedness training with operational metrics (service continuity, safety events, and staffing strain) to assess real readiness.

The co‐citation emphasis on nurses’ willingness to report to duty and perceived readiness further highlights that surge capacity depends on trust, protection, and role clarity—leadership responsibilities that shape attendance, morale, and retention during emergencies [19, 21, 30]. In pandemic contexts, the psychological burden documented in large‐scale studies and syntheses reinforces the need for leadership strategies that prioritize recovery, burnout prevention, and mental health support as preparedness measures, not merely post‐crisis responses [8, 27, 46]. Readiness, therefore, is demonstrated through sustained care delivery under pressure, supported by leadership that protects both workforce well‐being and service quality. This supports proactive leadership strategies such as early risk communication, fair deployment policies, and visible support systems that maintain workforce trust.

### 4.9. Implications for Future Research

Although methodological descriptors were excluded from the final substantive co‐word map, the keyword‐cleaning audit and broader corpus review indicate that much of the field still relies on self‐reported competence, survey‐based assessment, and cross‐sectional designs. While these designs are useful for rapid assessment, they limit causal inference and provide limited evidence on whether preparedness interventions improve workforce or patient outcomes. Future studies should prioritize longitudinal designs, intervention studies, and implementation research linking preparedness training, resilience supports, staffing strategies, and leadership interventions to measurable outcomes such as retention, burnout, absenteeism, care continuity, and patient safety [4, 14, 18, 20, 32, 46, 59]. Future research should also examine technology‐enabled preparedness, including telemedicine, telehealth, and virtual care, as emerging but still underdeveloped themes in the post‐2020 literature. Studies should assess not only whether telemedicine maintains service continuity during emergencies but also how it affects nurses’ workload, digital competence, communication burden, patient safety, and professional roles.

Future research should further examine how digital health technologies, artificial intelligence, and workforce analytics can strengthen nursing workforce preparedness and resilience. AI‐supported tools may be explored for predictive staffing, early detection of workforce strain, surge‐capacity modeling, allocation of emergency roles, and identification of units at risk for burnout, absenteeism, or reduced service continuity. Workforce analytics dashboards could integrate indicators such as staffing levels, skill mix, overtime, absenteeism, redeployment frequency, occupational exposure, mental health risk, turnover intention, and patient‐safety events. However, future studies should move beyond technical feasibility and evaluate whether these tools improve managerial decision‐making, staff well‐being, equitable deployment, care continuity, and emergency response capacity. Research should also examine ethical and implementation issues, including data privacy, transparency of AI‐supported decisions, algorithmic bias, staff trust, and the risk of increasing surveillance burden on nurses.

### 4.10. Integrative Implication: A Three‐Pillar Preparedness Model for Nursing

Viewed collectively, the bibliometric structure supports an integrated three‐pillar preparedness model: (1) competence and readiness (disaster nursing skills, confidence, and surge capability), (2) resilience and mental health protection (endurance, recovery, and burnout mitigation), and (3) system supports (staffing, safety culture, leadership, and education standards). This synthesis integrates foundational disaster competency frameworks [9, 10] with pandemic‐era evidence on workforce vulnerability [5, 45] and system‐level determinants of outcomes and sustainability [17, 24]. The model clarifies preparedness as a dynamic, multilevel construct rather than a static checklist. Positioned as a leadership agenda, the model supports measurable implementation targets across training, psychosocial safeguards, and structural readiness to sustain safe patient care under prolonged disruption.

### 4.11. Limitations and Future Works

Several limitations warrant consideration and point to directions for future research. First, the dataset was limited to Scopus‐indexed, English‐language journal articles and reviews. Although Scopus was selected to ensure consistent bibliometric metadata for co‐citation, co‐word, collaboration, thematic evolution, and hotspot analyses, reliance on a single database may introduce coverage bias. Relevant studies indexed only in Web of Science, PubMed, CINAHL, or other discipline‐specific databases may not have been captured. In addition, policy documents, technical reports, guidelines, books, and gray literature may be underrepresented despite their importance in emergency preparedness and workforce planning. Database triangulation was not undertaken because merging multiple databases would require extensive deduplication and normalization of cited references, author names, source titles, keywords, and affiliations, which may affect the stability of bibliometric network structures. Future bibliometric reviews should compare Scopus with Web of Science, PubMed, CINAHL, and relevant gray literature sources to test the robustness of the present findings and provide a broader representation of nursing workforce preparedness scholarship.

Second, bibliometric analyses are inherently dependent on metadata quality and analytic parameter choices, including search‐string construction, threshold settings, normalization procedures, and clustering algorithms. These decisions can influence network structures, thematic boundaries, and hotspot detection. Although transparent and reproducible procedures were applied, alternative search strings, database combinations, or VOSviewer parameter settings may yield slightly different structural representations of the field. Future studies may conduct sensitivity analyses using alternative thresholds, compare outputs across databases, and triangulate bibliometric findings with qualitative, expert‐driven, or policy‐oriented approaches to strengthen interpretation.

Third, the strong dominance of COVID‐19–related terms and references should be interpreted cautiously. Because the pandemic generated an unusually large and rapidly cited body of literature, the prominence of COVID‐19 in the co‐citation, co‐word, and hotspot analyses may partly reflect pandemic‐era citation concentration, publication acceleration, and recency effects. Therefore, COVID‐19–related nodes and keywords may overrepresent short‐term scholarly attention compared with older disaster preparedness themes that accumulated influence more gradually. At the same time, the co‐occurrence of COVID‐19 with resilience, burnout, moral distress, mental health, telemedicine, staffing, safety, and service continuity suggests that the pandemic also contributed to a more stable shift in how nursing workforce preparedness is conceptualized. Future bibliometric studies should revisit the field after the pandemic publication surge stabilizes and should compare pre‐pandemic, acute‐pandemic, and post‐pandemic periods to determine which COVID‐19–related themes remain enduring components of nursing preparedness scholarship.

Finally, bibliometric methods are descriptive and relational in nature. While they effectively identify intellectual foundations, thematic trajectories, and emerging hotspots, they do not establish causal relationships or assess the effectiveness of preparedness interventions. Future research should therefore prioritize longitudinal and intervention‐focused designs that empirically link preparedness strategies with workforce outcomes, service continuity, and patient safety indicators. Incorporating foresight‐oriented methods, such as scenario analysis, Delphi studies, and futures literacy frameworks, would further strengthen the field’s capacity to anticipate and proactively respond to emerging health crises.

Future bibliometric and empirical studies should also track the growth of AI‐enabled workforce planning, digital preparedness platforms, telehealth‐supported emergency care, and workforce analytics as emerging domains in nursing management scholarship. These areas may define the next phase of research on preparedness by linking bibliometric trends with measurable workforce, organizational, and patient‐care outcomes.

## 5. Conclusion

This bibliometric thematic evolution and hotspot analysis provides a time‐resolved map of nursing workforce preparedness scholarship from 2000 to 2025. Across a final retained corpus of 7570 Scopus‐indexed articles and reviews, composed of 6950 articles and 620 reviews, the field demonstrates sustained growth, broad international participation, and a mature citation structure, confirming preparedness as a stable and influential domain of nursing research. Rather than converging on a single definition, the literature reflects preparedness as a multidimensional construct in which competence, resilience, and system readiness co‐develop in response to recurring crises.

The field’s intellectual foundations are practice‐driven and crisis‐responsive, anchored in disaster preparedness competence research and reinforced by psychological resilience and mental health scholarship that intensified during the COVID‐19 pandemic. Normative frameworks and professional standards shape how preparedness is operationalized, while workforce structure and safety research emphasize preparedness as a system property dependent on staffing adequacy, safety culture, and organizational support.

Co‐word clustering and thematic evolution reveal a clear temporal shift from early emergency response functions to training and system readiness and more recently toward workforce well‐being, coping, burnout, and adaptive service continuity. Hotspot indicators confirm that contemporary preparedness scholarship increasingly integrates psychosocial sustainability alongside competence and system design. Collectively, these findings provide an evidence‐anchored framework to guide educators, leaders, and policymakers in strengthening nursing workforce preparedness by aligning competence development, mental health protection, and structural readiness to sustain safe care delivery under prolonged disruption. For nursing management, the findings support a shift from episodic disaster training toward strategic, data‐informed workforce preparedness that integrates policy, leadership, organizational capacity, digital innovation, and sustained protection of nurses’ well‐being.

## Author Contributions

The lead authors conceived the review, developed the search strategy, and oversaw the bibliometric and critical analyses. Assigned team members conducted database searching, screening, data extraction, and initial mapping. All authors contributed to interpretation and drafting and revision of the manuscript.

## Funding

This work received no specific funding from public, commercial, or nonprofit funding bodies.

## Disclosure

All authors approved the final submission.

## Ethics Statement

As this study is a bibliometric and critical review of published literature and involved no human participants, identifiable data, or interventions, formal ethics committee approval was not required. Nonetheless, ethical standards were observed through accurate source representation, avoidance of duplicate counting, and cautious interpretation of bibliometric and conceptual findings.

## Conflicts of Interest

The authors declare no conflicts of interest.

## Supporting Information

Additional supporting information can be found online in the Supporting Information section.

## Supporting information


**Supporting Information** Supporting Table S1. Leading journals publishing research on nursing workforce preparedness in health emergencies (2000–2025). Supporting Table S2. Top international country collaboration links in nursing workforce preparedness research. Supporting Table S3. Country‐attributed publication counts and international collaboration patterns (SCP vs. MCP) in nursing workforce preparedness research. Supporting Table S4. Periodized top author keywords and thematic evolution in nursing workforce preparedness research (2000–2025). Supporting Table S5. Emerging hotspot keywords in nursing workforce preparedness research (2020–2025 vs. 2010–2019). Supporting Table S6. Top productive authors in nursing workforce preparedness research. Supporting Table S7. Top institutional contributors in nursing workforce preparedness research. Supporting Table S8. Co‐word keyword harmonization and exclusion audit for the final VOSviewer map. Supporting Figure S1. PRISMA‐style workflow of study identification and selection.

## Data Availability

The bibliometric data for this review were obtained from the Scopus database. Processed datasets (e.g., included‐article lists and aggregated network metrics) may be requested from the corresponding author, subject to database licensing and access restrictions.
