# Supplementary material for: Nursing Workforce Preparedness and Resilience in Health Emergencies (2000–2025): A Bibliometric Review of Thematic Evolution and Emerging Hotspots
Source: J Nurs Manag. 2026 Jul 29;2026:7758497. doi: 10.1155/jonm/7758497 (PMC13420352; doi:10.1155/jonm/7758497)
Supplement: Supplementary file 1 — Supporting Information Supporting Table S1. Leading journals publishing research on nursing workforce preparedness in health emergencies (2000–2025). Supporting Table S2. Top international country collaboration links in nursing workforce preparedness research. Supporting Table S3. Country‐attributed publication counts and international collaboration patterns (SCP vs. MCP) in nursing workforce preparedness research. Supporting Table S4. Periodized top author keywords and thematic evolution in nursing workforce preparedness research (2000–2025). Supporting Table S5. Emerging hotspot keywords in nursing workforce preparedness research (2020–2025 vs. 2010–2019). Supporting Table S6. Top productive authors in nursing workforce preparedness research. Supporting Table S7. Top institutional contributors in nursing workforce preparedness research. Supporting Table S8. Co‐word keyword harmonization and exclusion audit for the final VOSviewer map. Supporting Figure S1. PRISMA‐style workflow of study identification and selection. [file JONM-2026-7758497-s001.docx]

**Supplementary Table S1. Leading Journals Publishing Research on Nursing Workforce Preparedness in Health Emergencies (2000–2025)**

| **Journal** | **Publications** | **Total citations** | **Avg citations/paper** |
| --- | --- | --- | --- |
| Journal of Emergency Nursing | 216 | 2,952 | 13.67 |
| International Emergency Nursing | 151 | 2,454 | 16.25 |
| BMC Nursing | 132 | 1,028 | 7.79 |
| Journal of Clinical Nursing | 123 | 3,476 | 28.26 |
| Journal of Advanced Nursing | 111 | 3,422 | 30.83 |
| International Journal of Environmental Research and Public Health | 110 | 2,444 | 22.22 |
| PLOS ONE | 107 | 1,698 | 15.87 |
| Disaster Medicine and Public Health Preparedness | 102 | 875 | 8.58 |
| BMJ Open | 99 | 1,491 | 15.06 |
| International Nursing Review | 93 | 2,073 | 22.29 |

Note. Ranked by number of publications in the retained corpus. Total citations were computed from the Scopus “Cited by” field in the retained CSV.

**Supplementary Table S2. Top International Country Collaboration Links in Nursing Workforce Preparedness Research**

| **Country A** | **Country B** | **Coauthored papers** |
| --- | --- | --- |
| Canada | United States | 66 |
| Australia | United Kingdom | 63 |
| United Kingdom | United States | 60 |
| Australia | United States | 55 |
| China | Hong Kong | 33 |
| Switzerland | United States | 29 |
| China | United States | 27 |
| Canada | United Kingdom | 27 |
| Australia | Canada | 25 |
| Japan | United States | 25 |

Note. Country pairs were counted once per paper when at least two countries were extracted from the affiliation metadata. Countries were parsed from the Scopus affiliation fields

**Supplementary Table S3. Country-attributed publication counts and international collaboration patterns (SCP vs. MCP) in nursing workforce preparedness research**

| **Country** | **Country-attributed publications** | **Share*** | **SCP** | **MCP** | **MCP share** |
| --- | --- | --- | --- | --- | --- |
| United States | 2534 | 26.9% | 2063 | 471 | 18.6% |
| United Kingdom | 753 | 8.0% | 491 | 262 | 34.8% |
| Australia | 636 | 6.7% | 401 | 235 | 36.9% |
| Canada | 442 | 4.7% | 278 | 164 | 37.1% |
| China | 393 | 4.2% | 286 | 107 | 27.2% |
| Sweden | 238 | 2.5% | 151 | 87 | 36.6% |
| Iran | 215 | 2.3% | 168 | 47 | 21.9% |
| Turkey | 190 | 2.0% | 160 | 30 | 15.8% |
| Italy | 180 | 1.9% | 106 | 74 | 41.1% |
| Saudi Arabia | 179 | 1.9% | 83 | 96 | 53.6% |

Note. *Share was computed among country-publication instances with extractable affiliation-country data (n = 9,425), not unique papers. Because multi-country publications were counted once for each participating country, the total number of country-publication instances exceeds the final retained corpus of 7,570 documents. SCP = single-country publications; MCP = multi-country publications. MCP share refers to the proportion of a country’s publications that involved international collaboration.

Supplementary Table S4. Periodized top author keywords and thematic evolution in nursing workforce preparedness research (2000–2025)

| **Time period** | **Rank** | **Top author keywords (descending prominence)** | **Frequency** |
| --- | --- | --- | --- |
| **2000-2009 (retained n=981; keyword-bearing n=432)** | 1 | emergency nursing | 28 |
|  | 2 | emergency department | 27 |
|  | 3 | triage | 24 |
|  | 4 | resuscitation | 19 |
|  | 5 | disaster response | 17 |
|  | 6 | mass casualty | 16 |
|  | 7 | emergency preparedness | 15 |
|  | 8 | trauma care | 14 |
|  | 9 | acute care | 14 |
|  | 10 | disaster management | 12 |
| **2010-2019 (retained n=1989; keyword-bearing n=1502)** | 1 | emergency department | 107 |
|  | 2 | simulation | 104 |
|  | 3 | education | 85 |
|  | 4 | disaster | 75 |
|  | 5 | training | 59 |
|  | 6 | emergency nursing | 58 |
|  | 7 | nursing education | 49 |
|  | 8 | emergency medicine | 49 |
|  | 9 | disaster preparedness | 44 |
|  | 10 | triage | 44 |
| 2020-2025 (retained n=4600; keyword-bearing n=4014) | 1 | COVID-19 | 1,155 |
|  | 2 | resilience | 391 |
|  | 3 | pandemic | 250 |
|  | 4 | burnout | 186 |
|  | 5 | qualitative research | 166 |
|  | 6 | mental health | 166 |
|  | 7 | emergency department | 159 |
|  | 8 | education | 140 |
|  | 9 | COVID-19 pandemic | 139 |
|  | 10 | nursing education | 124 |

Note. Author keywords were split on semicolons, normalized for case and common COVID-19 variants, and counted once per paper. Generic indexing and demographic terms were excluded. Frequency refers to the number of papers in the period containing the keyword. Author-keyword thematic evolution was analyzed separately from the VOSviewer co-word conceptual map.

**Supplementary Table S5. Emerging hotspot keywords in nursing workforce preparedness research (2020–2025 vs. 2010–2019)**

| **Keyword** | **2010–2019** | **2020–2025** | **Δ (20–25 minus 10–19)** | **Total (2000–2025)** | **APY (mean year)** |
| --- | --- | --- | --- | --- | --- |
| COVID-19 | 0 | 1,155 | 1,155 | 1,155 | 2022.48 |
| resilience | 29 | 391 | 362 | 420 | 2022.47 |
| pandemic | 11 | 250 | 239 | 265 | 2021.78 |
| burnout | 9 | 186 | 177 | 195 | 2022.71 |
| mental health | 21 | 166 | 145 | 191 | 2021.70 |
| qualitative research | 22 | 166 | 144 | 193 | 2022.07 |
| COVID-19 pandemic | 0 | 139 | 139 | 139 | 2022.92 |
| healthcare workers | 7 | 108 | 101 | 117 | 2022.09 |
| anxiety | 5 | 96 | 91 | 101 | 2022.34 |
| stress | 10 | 100 | 90 | 113 | 2021.44 |
| depression | 2 | 81 | 79 | 84 | 2022.17 |
| disaster preparedness | 44 | 121 | 77 | 179 | 2020.19 |
| nursing education | 49 | 124 | 75 | 182 | 2020.31 |
| preparedness | 38 | 106 | 68 | 159 | 2019.42 |
| nursing students | 15 | 83 | 68 | 102 | 2021.19 |
| leadership | 14 | 81 | 67 | 100 | 2021.10 |
| pandemics | 2 | 69 | 67 | 71 | 2022.20 |
| psychological resilience | 1 | 62 | 61 | 63 | 2022.81 |
| moral distress | 2 | 47 | 45 | 49 | 2022.71 |
| telehealth | 3 | 37 | 34 | 40 | 2022.42 |
| telemedicine | 8 | 30 | 22 | 38 | 2021.03 |
| virtual care | 0 | 7 | 7 | 7 | 2023.71 |

Note. Δ indicates the increase in keyword frequency from 2010–2019 to 2020–2025. APY = average publication year across retained papers containing the keyword. The table prioritizes high-growth terms and includes technology-enabled care terms relevant to telemedicine. Methodological author keywords such as qualitative research are reported as emerging research-practice or methodological trends, but they were not interpreted as substantive conceptual domains in the final co-word map.

Supplementary Table S6. Top productive authors in nursing workforce preparedness research

| **Rank** | **Author** | **Publications** | **First-author papers** | **Total citations** | **Average citations/paper** |
| --- | --- | --- | --- | --- | --- |
| 1 | Considine, Julie | 29 | 16 | 772 | 26.62 |
| 2 | Veenema, Tener Goodwin | 29 | 14 | 866 | 29.86 |
| 3 | Wolf, Lisa Adams | 18 | 13 | 227 | 12.61 |
| 4 | Lindström, Veronica | 15 | 1 | 197 | 13.13 |
| 5 | Labrague, Leodoro J. | 14 | 12 | 1,725 | 123.21 |
| 6 | Rebmann, Terri L. | 14 | 13 | 399 | 28.50 |
| 7 | Cooper, Simon Jr. | 14 | 4 | 467 | 33.36 |
| 8 | Rushton, Cynda Hylton | 13 | 5 | 411 | 31.62 |
| 9 | Ranse, Jamie C. | 13 | 7 | 346 | 26.62 |
| 10 | Hammad, Karen S. | 13 | 3 | 727 | 55.92 |

Note. Author productivity was calculated from the Scopus “Author full names” field in the retained CSV. Publication counts were counted once per author per paper. Total citations were computed from the Scopus “Cited by” field.

Supplementary Table S7. Top institutional contributors in nursing workforce preparedness research

| **Rank** | **Institution / affiliation organization** | **Publications** | **Total citations** |
| --- | --- | --- | --- |
| 1 | Monash University | 93 | 2,509 |
| 2 | University of California | 87 | 2,266 |
| 3 | Karolinska Institutet | 75 | 1,294 |
| 4 | University of Toronto | 67 | 1,664 |
| 5 | Johns Hopkins School of Nursing | 59 | 1,524 |
| 6 | Harvard Medical School | 56 | 1,656 |
| 7 | Deakin University | 53 | 1,456 |
| 8 | Johns Hopkins University School of Medicine | 52 | 2,114 |
| 9 | The University of British Columbia | 47 | 1,228 |
| 10 | University of Pennsylvania | 47 | 1,684 |
| 11 | McMaster University | 44 | 1,164 |
| 12 | University of Technology Sydney | 41 | 905 |
| 13 | King Saud University | 41 | 488 |
| 14 | The University of Queensland | 40 | 946 |
| 15 | Johns Hopkins Bloomberg School of Public Health | 39 | 1,348 |

Note. Institutional contributions were calculated from the Scopus affiliation metadata in the retained CSV. Institutions were counted once per paper when present in the affiliation field. Generic department-only labels were excluded where a parent institution could be identified.

**Supplementary Table S8**. Co-word keyword harmonization and exclusion audit for the final VOSviewer map

| **Original keyword / descriptor** | **Final treatment** | **Rationale** |
| --- | --- | --- |
| nurse; nurses; nursing | Harmonized as nursing / nurses | Consolidated singular, plural, and lexical variants referring to the nursing workforce. |
| COVID-19; coronavirus disease 2019; SARS-CoV-2; severe acute respiratory syndrome coronavirus 2 | Harmonized as COVID-19 | Consolidated alternative labels referring to the COVID-19 pandemic context. |
| pandemic; pandemics | Harmonized as pandemic(s) | Consolidated singular and plural variants. |
| health personnel; health care personnel | Harmonized as health care personnel | Consolidated closely related workforce descriptors. |
| psychological resilience; resilience, psychological; resilience | Interpreted within the resilience domain | Consolidated resilience-related variants for conceptual interpretation. |
| questionnaire; questionnaires; surveys and questionnaires | Excluded from substantive co-word interpretation | Treated as instrument or measurement descriptors, not substantive preparedness concepts. |
| cross-sectional study; cross-sectional studies | Excluded from substantive co-word interpretation | Treated as study-design descriptors, not substantive preparedness concepts. |
| qualitative research; interview; review | Excluded from substantive co-word interpretation | Treated as methodological descriptors in the co-word map, not conceptual domains. |
| methodology; practice guideline | Excluded from substantive co-word interpretation | Treated as methodological or document-type descriptors. |
| standard; standards | Excluded from substantive co-word interpretation | Treated as broad indexing descriptors. |
| human; humans; female; male; adult; aged; middle aged; young adult | Excluded from substantive co-word interpretation | Treated as demographic indexing descriptors. |
| article; priority journal; controlled study; major clinical study; human experiment; procedures; physician | Excluded from substantive co-word interpretation | Treated as generic Scopus indexing or non-conceptual descriptors. |

Note. This audit documents the thesaurus-based harmonization and exclusion decisions used for the final co-word analysis. Methodological, demographic, and database-indexing descriptors were excluded from the substantive conceptual interpretation to avoid treating study designs, instruments, or indexing labels as domains of nursing workforce preparedness.

**
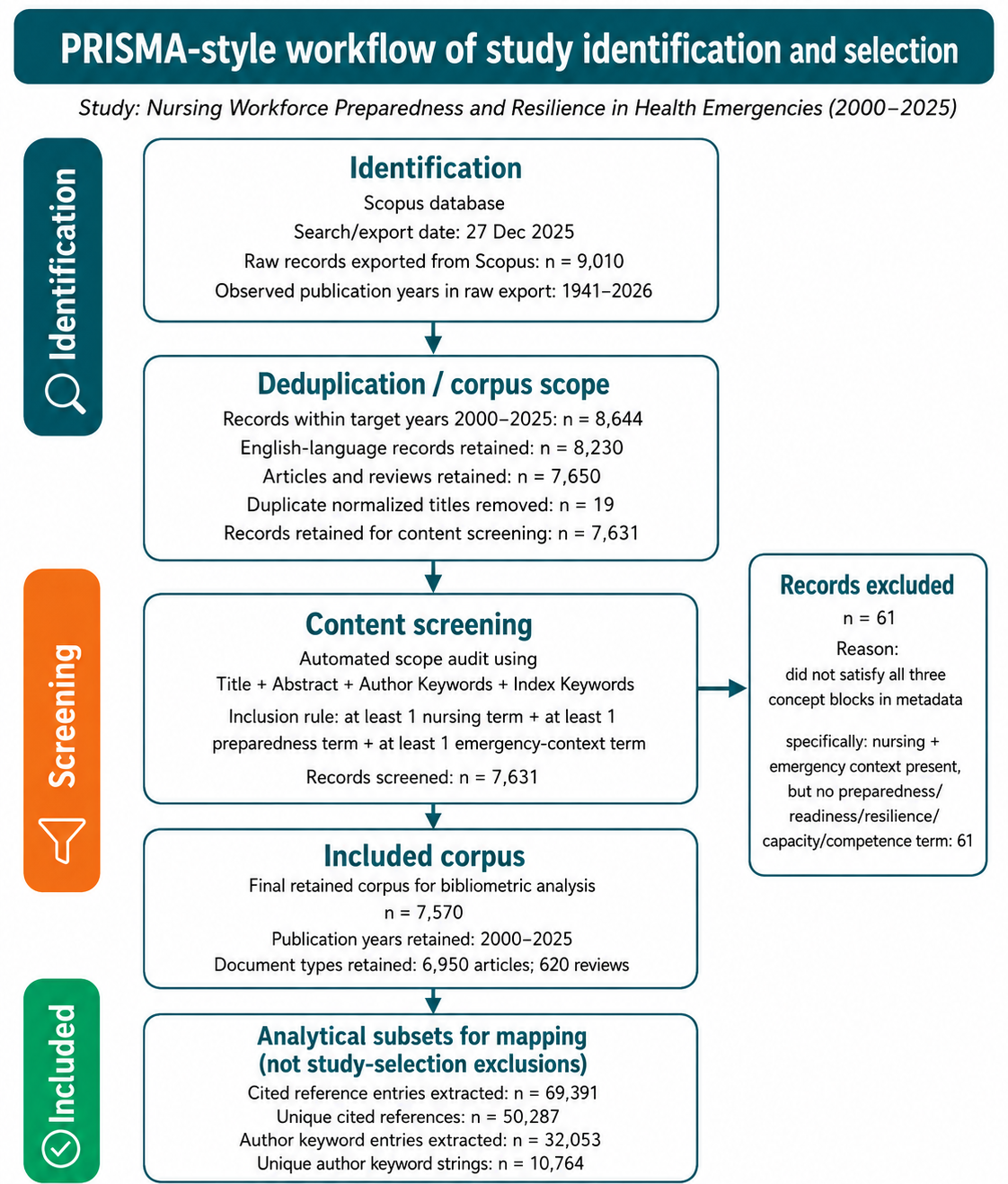
Supplementary Figure S1. PRISMA-style workflow of study identification and selection**

**Note.** The workflow summarizes construction of the final retained corpus used for VOSviewer mapping and supplementary analyses.
